# A sensitive method to determine ^210^Po and ^210^Pb in environmental samples by alpha spectrometry using CuS micro-precipitation

**DOI:** 10.1038/s41598-023-46230-9

**Published:** 2023-11-13

**Authors:** Stephanie Walsh, Matthew J. Bond, Nicolas Guérin, Jules M. Blais, David J. Rowan

**Affiliations:** 1 Canadian Nuclear Laboratories, Chalk River Laboratories, Chalk River, ON K0J 1J0 Canada; 2 Department of Biology, University of Ottawa, Ottawa, ON K1N 6N5 Canada

**Keywords:** Chemistry, Analytical chemistry, Environmental sciences, Environmental chemistry

## Abstract

A new sensitive method to determine polonium-210 (^210^Po) and lead-210 (^210^Pb) in a diversity of environmental samples was developed. For fresh and marine waters, Po was pre-concentrated using a titanium (III) hydroxide (Ti(OH)_3_) co-precipitation. Solid environmental samples were digested with nitric acid (HNO_3_) and hydrogen peroxide (H_2_O_2_). The alpha thin layer source was prepared using CuS micro-precipitation and ^210^Po was measured by alpha spectrometry. Lead-210 was left to decay for up to a year and indirectly measured via its progeny, ^210^Po. The chemical recoveries for ^210^Po and ^210^Pb were high, 90% and 97%, respectively, for a large variety of samples and a very low minimum detectable activity (MDA) was obtained. The method was validated using standardized solutions and certified reference materials.

## Introduction

Polonium-210 (^210^Po) is a naturally occurring alpha-emitting radioisotope from the uranium-238 (^238^U) decay series with a high activity per unit mass (1.66 × 10^14^ Bq g^−1^) and a relatively short radioactive half-life (t_1/2_ = 138.4 days)^1^, making it one of the most toxic radionuclides in the environment^2–4^. Lead-210 (^210^Pb) (t_1/2_ = 22.2 years)^1^ is a beta-emitting radioisotope that decays to bismuth-210 (^210^Bi) (t_1/2_ = 5.0 days)^1^, and then to ^210^Po. Lead-210 and ^210^Po are linked by their radiogenic association and can be in secular equilibrium in static environments or unsupported by each other in dynamic conditions^2,4,5^. Naturally, ^210^Po and ^210^Pb activities could be more elevated in some regions based on environmental characteristics such as local geology, land cover or forest fires^6^. These radionuclides can also be anthropogenically enriched by activities such as mining, fracking and fossil fuel combustion, which bring ^238^U and its decay products to the earth’s surface and surrounding environments^7–9^.

Despite the high toxicity and significant contribution of ^210^Po to the natural radiation dose received by living organisms (> 90%)^10,11^, ^210^Po remains the least studied natural radionuclide in freshwater environments^5,12,13^. This is mainly because some of its compounds are volatile and there are limited rapid methods to measure it in low-level environmental samples containing a high amount of organic matter such as animal tissues, foodstuff and vegetation^12,14^. Traditionally, ^210^Po has been determined using the spontaneous deposition technique where ^210^Po is self-plated on a metallic disc in an acidic solution^13,15^. This method while effective for some applications, has limitations for us in large-scale environmental studies. These limitations include high sample throughput times, detection limits not suitable for all environmental samples and the possibility of interference of ions, such as iron and chromium, with the Po plating^16–18^. Conversely, ^210^Pb has been well studied in several environmental samples^19,20^. Measurements of ^210^Pb can be efficiently determined by gamma spectrometry in solid sample matrices if a large amount of sample is available and/or if the activity concentrations are high enough. However, the determination of trace activities of ^210^Pb in certain matrices and small size samples, such as water and freshwater organisms, can be labour intensive, expensive and sometimes impossible due to detection limits that are too high^21^.

Without adequate methods to measure environmental levels of ^210^Pb and ^210^Po, there is a significant lack of data on the occurrence of these radionuclides in the environment; thus, insufficient data are available to understand their behaviour in the environment The current lack of understanding on the activities and movement of ^210^Pb and ^210^Po in the environment, combined with increased industrial activities that can elevate their concentrations and mobility in ecosystems such as uranium mining; as well as natural events, such as forest fires underscores the importance of developing a simple and rapid method to determine these radionuclides to support robust and reliable environmental studies and thereby generate more accurate risk assessments for human and non-human biota^11,14^.

A more efficient and accurate method was developed by Guérin and Dai^22,23^ to prepare thin alpha layer sources of Po using copper sulfide (CuS) micro-precipitation. This method consists of selectively co-precipitating polonium sulfide (PoS) with CuS from other potential interferences in an acidic solution. This method had only been used for simple matrices such as drinking water and not for more complex environmental samples, such as biological tissues. To expand the applicability of this method, a novel method to determine ^210^Po and, indirectly, ^210^Pb, in a variety of abiotic (fresh and marine waters) and biotic (freshwater organisms and vegetation) environmental samples is presented. The recovery was optimized, the MDA was determined, and the method was validated.

## Materials and methods

### Reagents and standards

All chemicals used were analytical grade except acids, which were trace metal grade. Ultrapure water (UPW) from a Milli-Q water system (Millipore, Bedford MA, USA) was used for the preparation of all reagents and standards. Hydrochloric acid (HCl), nitric acid (HNO_3_), hydrogen peroxide (H_2_O_2_), sodium hydroxide (NaOH), copper chloride (CuCl_2_·2H_2_O), and ethanol (CH_3_CH_2_OH) were purchased from Fisher Scientific (Fair Lawn, NJ). Titanium trichloride (TiCl_3_) and sodium sulfide (Na_2_S·9H_2_O) were obtained from Millipore Sigma (St. Louis, MO).

Certified standard solutions of ^210^Po, ^209^Po and ^210^Pb were purchased from Eckert & Ziegler Isotope Products (Valencia, CA). In addition, reference materials (CLV1 and CLV2) containing ^210^Po and ^210^Pb, in equilibrium, were purchased from the International Atomic Energy Agency (IAEA; Vienna, Austria)^24^. The accuracy of the certified reference materials was tested by an inter-laboratory program established by Natural Resources Canada^25^. A certified stable lead standard was purchased from Millipore Sigma (Oakville, ON).

### Analytical equipment

Radiological activity concentrations (^209^Po, ^210^Po and ^210^Pb) were detected using an Alpha Analyst alpha spectrometer (Mirion Technologies (Canberra), Meriden, CT) with a counting efficiency superior to 17% and a background of < 1 count/hour in the energy regions of interest. Apex Alpha counting productivity software was used for detector setup and control, quality assurance, sample analysis and elaboration. The Pb chemical yield was verified by inductively coupled mass plasma spectrometry (ICP-MS) (8900 ICP-MS Triple Quad, Agilent Technologies, Santa Clara, CA).

### Sample collection and preservation

#### Surface water

To test the applicability of this method for a range of water types, surface waters were collected from marine (n = 4) and fresh water (n = 13) systems representing a gradient of ionic content (Supplementary Fig. S1, Supplementary Fig S2 and Supplementary Table S1). Marine surface waters (4 L) were collected within the St. Lawrence Estuary and Gulf of St. Lawrence in eastern Canada. Freshwater samples (4 L) were collected from Perch Lake in Ontario,Canada located on the Chalk River Laboratories (CRL) site. Immediately following collection, samples were filtered using a 0.45 µm filter (FHT-45, Waterra, Mississauga, ON) and acidified to 1% HNO_3_. The electrical conductivity of the samples was measured using an Oakton 450 Meter (Thomas Scientific, Swedesboro, NJ), and the elemental content was measured by ICP-MS (Supplementary Table S2). The range of conductivity, calcium mass concentration, and chloride mass concentration for the samples collected are shown in Table 1.

**Table 1:** Aqueous sample properties.

	Conductivity µS cm^−1^	[Ca^2+^] mg L^−1^	[Cl^–^] mg L^−1^
Marine water	29,200–47,100	330–210	14,776–28,946
Fresh water	115–475	4.4–5.8	36–46

#### Biota

Biota from selected trophic levels were collected in the Lac Granet and Lac Camille-Roy systems (main lake and connected streams) of western Québec, Canada. Biotic samples collected included algae (n = 3), macrophytes (n = 7), plankton (n = 2), invertebrates (n = 9), molluscs (n = 6) and fish (n = 18). Plankton samples were collected with plankton nets (WildCo, Saginaw, MI) mesh sizes 153 µm and towed behind a boat. Algae, macrophytes, invertebrates and fish were collected using dip and kick nets (WildCo, Saginaw, MI). Immediately after collection, invertebrates and molluscs were placed in UPW and allowed to purge their gut contents for approximately 12 hours^26^. All samples were thoroughly rinsed in lake water and kept frozen until further processing.

### Procedure

#### Sample processing and digestion

The samples were processed within 30 days of collection to reduce ^210^Po loss from radioactive decay and ^210^Po ingrowth from ^210^Pb. For the water samples, a one litre subsample was weighed into a glass beaker and acidified to pH 2 using concentrated HNO_3_ to stabilize the sample and keep the Po in solution. Next, a known amount of ^209^Po yield tracer (approximately 15 mBq) and stable Pb standard (approximately 2.0 µg) was added. The sample was filtered again through a 0.22 µm polystyrene filter (Fisher Scientific, Fair Lawn, NJ) to remove remaining particulate matter and colloids.

Biota samples, were processed wet and were minced using a scalpel and scissors and homogenized before distributing 1 to 2 g (wet weight) into a 15 mL polypropylene digestion tube. A known amount of ^209^Po (approximately 15 mBq) was added to each sample before digestion. To digest the sample, 3.3 mL of concentrated HNO_3_ were added to the digestion tube and allowed to sit for approximately 1 h followed by the addition of 2.0 mL of 30% H_2_O_2_. The sample solution was then heated to 50 °C for 48 h. Once removed, the dissolved sample solution was allowed to cool and it was transferred to a 50 mL polypropylene conical tube (Cole-Parmer, Montreal, QC). The sample was centrifuged at 3500 rpm for 1.5 min. The supernate was transferred to a new 15 mL polypropylene conical tube (Cole-Parmer, Montreal, QC) and diluted to a volume of 10 mL with UPW.

#### Water samples pre-concentration

For the water samples only, Po was co-precipitated with Ti(OH)_3_ using 0.6 mL of 10% m v^−1^ TiCl_3_, and the pH was adjusted between 10 and 12 using about 30 mL of 40% mv^−1^ sodium hydroxide (NaOH) solution. The fresh and marine water samples were left aside for 1 and 4 h, respectively, which allowed the Ti(OH)_3_ to settle and to easily remove most of the supernate by decantation. The precipitate was then recovered by centrifugation, dissolved with 3.3 mL of concentrated HNO_3_ and diluted to a volume of 10 mL with UPW.

#### CuS microprecipitation

. The 10 mL, 5 mol l^−1^ HNO_3,_ supernate collected following the water pre-concentration and biota sample digestion, was filtered through a 0.1 µm Resolve™ filter (Eichrom Technologies Inc., Lisle, IL) using a multi-hole vacuum box and recovered in a 50 mL polypropylene conical tube. Then, 0.2 mL of 500 µg mL^−1^ Cu(II) solution was added and mixed, followed immediately by the addition of 0.2 mL of 10% m v^−1^ Na_2_S, at which point a visible (brown) colloidal precipitate formed. The sample was then filtered immediately through a 0.1 µm Resolve™ filter. After rinsing the filter with 1 to 2 mL of UPW, the filtrate was set aside for future ^210^Pb determination and replaced with a 50 mL polypropylene conical waste tube. The filter was rinsed with 1 to 2 mL of 80% ethanol. The precipitate retained on the filter was air-dried and mounted on a stainless steel disc (AF Murphy Die & Machine Co Inc., North Quincy, MA) for ^210^Po determination by alpha spectrometry. Water samples were counted for 48–96 h, while biota samples were counted for 24 to 48 h. The in situ ^210^Po activity concentrations were calculated by Eq. (1):

1 $${\mathrm{A}}_{\mathrm{Po}-210}\, \mathrm{in\, situ} (\mathrm{dpm})={\left[{{A}^{\prime}}_{Po-210 }\left(\mathrm{dpm}\right)- {{A}^{\prime}}_{Po-210 ingrowth}\right]} \times {e}^{-\lambda PoT}$$

where A’_Po-210_ is the ^210^Po activity at the time of extraction, A’_Po-210 ingrowth_ is a correction factor for ^210^Po loss as well as ^210^Po ingrowth from ^210^Pb decay from the time of sampling and extraction, λ_Po_ is the decay constant for ^210^Po and T is time between sample collection and extraction^27,28^.

#### ^210^Pb determination

Lead-210 activity concentrations were determined by measuring the ingrowth activity of its daughter, ^210^Po, using alpha spectrometry. Following the CuS micro-precipitation step, an additional aliquot of approximately 15 mBq of ^209^Po yield tracer and 2.0 µg of stable Pb carrier was added to the final filtrate. The sample was then left aside for at least 4 months to allow^210^Po ingrowth. Finally, the CuS micro-precipitation was repeated a second time and the ^210^Pb activity was calculated using Eq. (2):

2 $${\mathrm{A}}_{\mathrm{Pb}-210}\mathrm{in\, situ} (\mathrm{dpm})={A}_{mPo }\times \mathrm{exp}({\lambda }_{Pb}{t}_{2})/{n}_{c }[1-exp\left({-\lambda }_{Po}{t}_{1}\right)]$$

where A_Pb-210_ in situ is the activity concentration of the sample at the time of collection, A_mPo_ is the activity of ^210^Po at the time of the second CuS micro-precipitation, t_2_ is a correction factor for the decay of ^210^Pb from the time of sample collection and second extraction, t_1_ is a ingrowth factor for ^210^Po from the decay of ^210^Pb for time between first and second extraction, λ_Po_ and λ_Pb_ are decay constants for ^210^Po and ^210^Pb, respectively^27,28^. The *n*_c_ parameter is the Pb chemical yield calculated by applying the following Eq. (3):

3 $${Y}_{Pb}=\frac{{C}_{Pb}\times {V}_{1}}{{C}_{Pbcarrier}\times {V}_{2}} \times 100$$

where C_Pb_ is the lead concentration detected by ICP-MS; V_1_ is the volume collected from the filtrate; C_Pbcarrier_ is the concentration of the Pb carrier added; and V_2_ is the initial sample volume.

### Method development

The method was applied to a variety of environmental samples, which included different matrices and sample sizes to tests its applicability for large scale field studies. For water samples, an additional filtration step was added before the micro-precipitation step to improve the chemical resocvery. In addition, following the Ti(OH)_3_ co-precipitation of marine waters, a longer settling period was essential to improve accuracy. To assess the possible loss of Pb during the Ti(OH)_3_ co-precipitation and CuS micro-precipitation steps, a stable lead standard was added and an aliquot was taken before and after the extraction.

### Figures of merit

The MDA was determined by measuring ^210^Po and ^210^Pb in ten reagent blank samples following all the steps of the described method and using the Currie equation^28^ (4):

4 $$MDA= \frac{{k}^{2}+2\cdot k\cdot \sqrt{2\cdot B}}{T \cdot \varepsilon \cdot R\cdot V\cdot F}$$

where *k* is a constant (1.645) to reach the 95% confidence interval, B is the number of background counts for a defined time in seconds (T), ε is the counting efficiency, R is the chemical recovery, V is the volume in litres and F is a unit conversion factor which equals 10^−3^.

The method accuracy and precision were determined by measuring ^210^Po (n = 7) and ^210^Pb (n = 3) in water samples spiked with a known activity of ^210^Po and^210^Pb, in equilibrium, using the developed method. The relative bias (B_ri_) and the relative precision (S_B_) were calculated using Eqs. 5 and 6. ^29,30^:

5 $${B}_{ri}= \frac{{A}_{i}-{A}_{ai}}{{A}_{ai}}\times 100\%$$

6 $${S}_{B}= \frac{\sqrt{{\sum }_{i=1}^{N}({B}_{ri}-{B}_{r}}{)}^{2}}{N-1}\times 100\%$$

where A_i_ is the measured activity, A_ai_ is the added activity, B_r_ is the mean relative bias and N is the number of replicates.

The method was validated for solid samples by determining the ^210^Po and ^210^Pb activity concentration in certified reference materials CLV-1 and CLV-2. The data followed a normal distribution and therefore a Student’s *t*-test was used to evaluate differences in the ^210^Po and ^210^Pb activity concentrations measured in the certified reference materials by the inter-laboratory program and this method.

### Animal care

The institutional review board that reviewed and approved the capture, handling and euthanasia of fish for this study was the Wildlife Management Branch of the province of Québec’s Ministry of Forests, Wildlife and Parks (permit # 2021-07-14-070-08-GP). This approved and issued permit covered all aspects of fish collection, animal welfare and euthanasia methods. All methods were carried out in accordance with relevant guidelines and regulations. Euthanasia of fish was conducted following Guideline 113 of the Canadian Council on Animal Care guidelines on the care and use of fish in research, teaching and testing^31^. Specifically, fish euthanasia was conducted immediately after capture by swift blow to the head (destruction of brain tissue). Where relevant, all methods are reported in accordance with ARRIVE guidelines (https://arriveguidelines.org) for the reporting of animal experiments.

## Results and discussion

### Recovery optimization

Initially, a chemical recovery of 37 ± 10% was obtained for environmental fresh water samples, which was much lower than test samples prepared with UPW (~ 85%). The average recovery increased to 89.8 ± 12.5% when the samples were re-filtered before the CuS micro-precipitation step using a 0.22 µm polyethersulfone filter (Fisher Scientific, Fair Lawn, NJ) to remove suspended particulate matter and colloids smaller than 0.45 µm in size. During the final filtration step, following the CuS micro-precipitation, the PoS precipitate as well as any particulate matter would be retained leading to a poor spectral resolution and recovery. This additional filtration step substantially improved the chemical yield and spectral resolution for field water samples.

**Figure 1:**
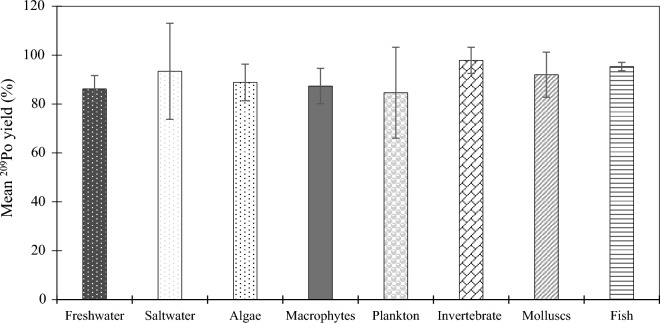
^209^Po recovery for initial ^210^Po extraction as a function of sample type. Error bars indicate the standard error of the mean.

Next, the method was tested on various types of samples and the results are shown in Fig. 1. The chemical recovery was high for all type of samples tested (90.7 ± 9.3%), which demonstrated that this method was versatile and robust.

**Figure 2:**
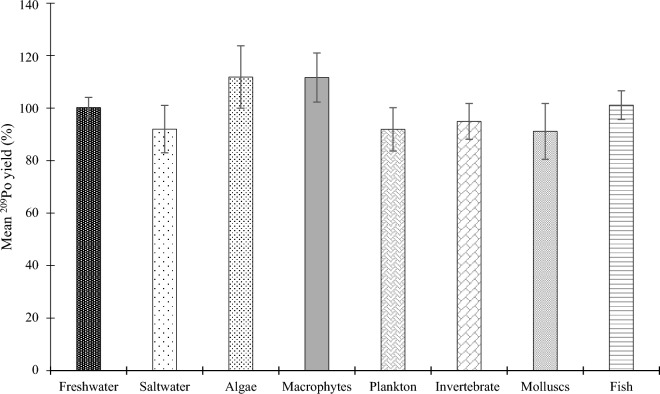
^209^Po recovery as a function of sample type for indirect ^210^Pb measurement via ^210^Po extraction following a 4 month in growth period. Error bars indicate the standard error of the mean.

Approximately 4 months after the initial ^210^Po extractions, the same samples were analyzed again to indirectly measure the ^210^Pb present in the sample. The chemical recovery following the ingrowth period was high (97.1 ± 8.2%) and consistent for all sample types as shown in Fig. 2.

### Figures of merit

**Figure 3:**
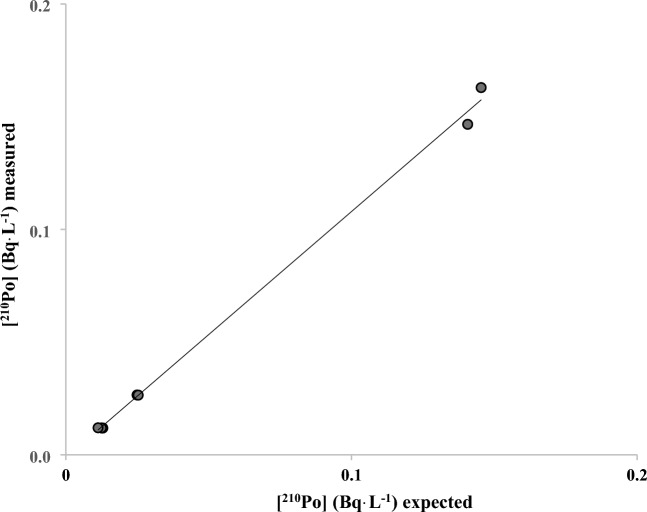
^210^Po recovery results for spiked water samples after CuS micro-precipitation.

**Figure 4:**
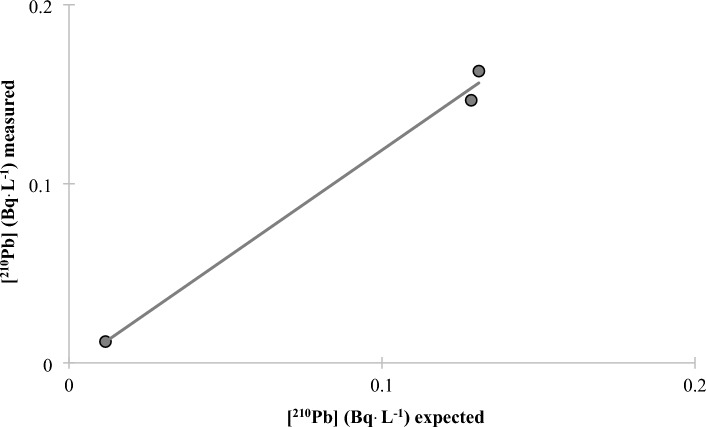
^210^Pb recovery results for spiked water samples following a 4 month in-growth period.

**Figure 5:**
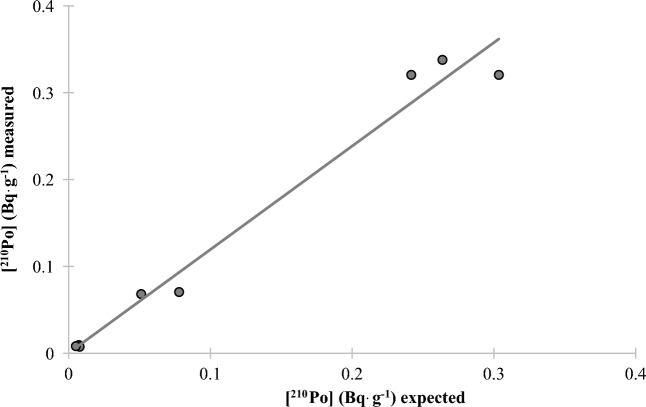
^210^Po recovery results for certified vegetation standards after CuS micro-precipitation.

**Figure 6:**
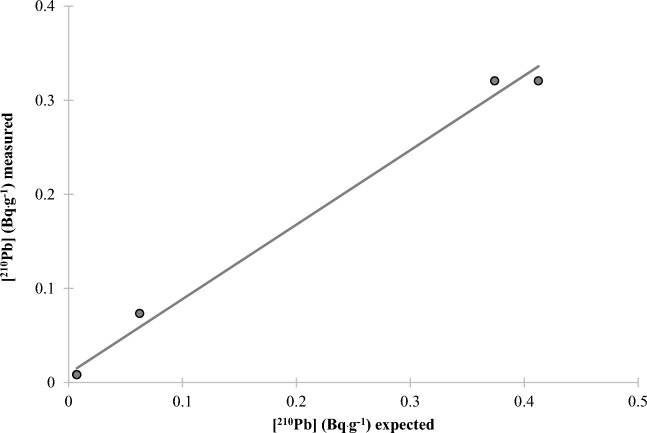
^210^Pb recovery results for certified vegetation standards after CuS micro-precipitation.

A very low MDA of 0.05, 0.02, and 0.01 mBq L^−1^ was obtained for 24, 48, and 96 h of counting, respectively (Table 2) The method was validated for water samples using water samples spiked with ^210^Po and ^210^Pb with a range of activities from 12 to 160 mBq and counted for 24, 48 and 96 h (Fig. 3). The mean ^210^Po chemical recovery was 97 ± 7% over the 3 count times. Three samples were allowed to sit for approximately 4 months when the CuS micro-precipitation was repeated to validate the method for ^210^Pb determination (Fig. 4) (mean ^210^Pb chemical recovery was 102 ± 7% and mean stable Pb chemical recovery was 108 ± 6%). To further validate the method for solid samples, two certified reference materials (CLV-1 and CLV-2) with a range of ^210^Po and ^210^Pb activities from 74 to 660 mBq g^−1^ were chosen to represent a range of activity concentrations. Mean ^210^Po chemical recoveries of 89 ± 3% and 80 ± 6% were calculated from the analyses of the two vegetation reference materials CLV-1 (n = 5) and CLV-2 (n = 4), respectively. A mean ^210^Pb chemical recovery of 101 ± 9. % was calculated from the analyses of the vegetation reference material CLV-1 (n = 5) (Table 2 and Figs. 5, 6). Furthermore, the comparison of the expected and measured ^210^Po and ^210^Pb activities in reference materials using a two-tailed Student’s *t*-test, assuming unequal variances (*p*-values > 0.05), indicates repeatability, confirming the method to be accurate for ^210^Po and ^210^Pb determination in environmental abiotic and biotic non-water samples. These results provided evidence that complex matrices, as well as reduced sample size and digestion temperatures, produced consistent and high polonium and lead recoveries (78–101%) (Table 3). The developed method was also accurate and precise as evidenced by the mean relative bias and mean relative precision presented in Table 2.

**Table 2:** Figure of merit for water method.

Counting time	24 h	48 h	96 h
Water standards			
MDA (mBq L^−1^) (n = 10)	0.05	0.02	0.01
Mean ^210^Po chemical recovery (%) for spiked water (n = 7)	93.4 ± 2.0	106.5 ± 2.4	94.8 ± 0.9
Mean ^210^Po relative bias (%)	− 6.9	6.5	− 5.2
^210^Po relative precision (%)	3.5	3.3	1.2
Statistics t-test (^210^Po)		t _(7, 0.05)_ = 2.18 *p* = 0.46	
Mean ^210^Pb chemical recovery (%) for spiked water (n = 3)		88.7 ± 8.9	
Mean ^210^Pb relative bias (%)		− 11.3	
^210^Pb relative precision (%)		8.9	
Statistics t-test (^210^Pb)		t _(3, 0.05)_ = 2.78 *p* = 0.80

**Table 3:** Figures of merit for solid environmental samples.

	CLV-1 (*n* = 5)	CLV-2 (*n* = 4)
IAEA vegetation standards		
Certified ^210^Po and ^210^Pb activity concentration (mBq g^−1^)	660 ± 60	74 ± 7
Mean ^210^Po recovery (%)	86.7 ± 7.0	78.5 ± 9.0
Mean^210^Po relative bias (%)	− 13.24	− 21.5
Mean ^210^Po relative precision (%)	8.0	9.0
Statistics t test (^210^Po)	t_(5, 0.05)_ = 2.78 *p* = 0.15	t_(4, 0.05)_ = 2.45 *p* = 0.07
Mean stable Pb recovery (%)	101.2 ± 9.0	
Mean ^210^Pb relative bias (%)	1.2	
Mean ^210^Pb relative precision (%)	7.4	
Students t test (^210^Pb)	t_(5, 0.05)_ = 2.30 *p* = 0.82	

### Application and comparison of the method

**Figure 7:**
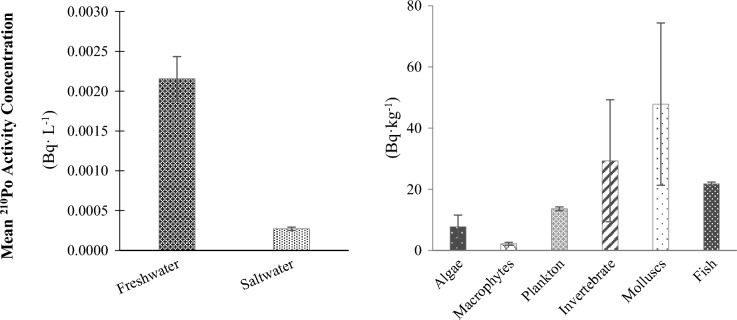
Mean ^210^Po activity concentrations measured for water samples and solid samples. Error bars indicate the standard error of the mean.

**Figure 8:**
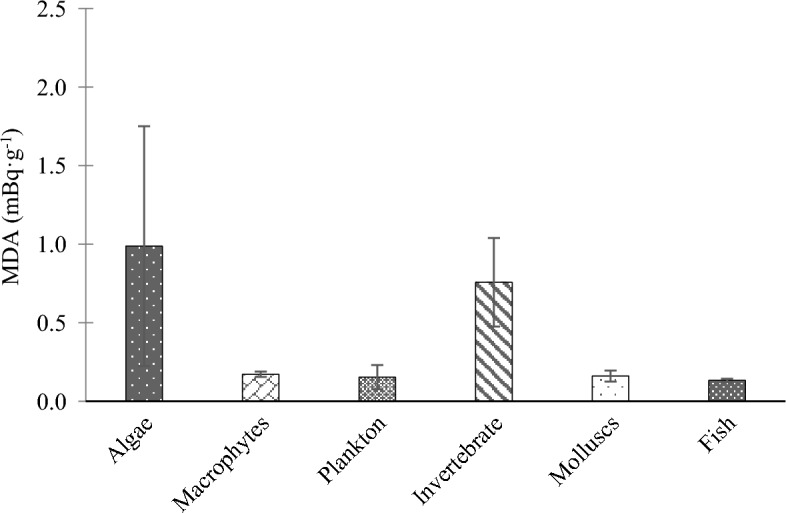
Mean MDAs measured for water samples and solid samples. Error bars indicate the standard error of the mean.

The mean activity concentrations of ^210^Po measured in different types of waters (freshwater and saltwater) and several types of freshwater organisms are shown in Fig. 7. Freshwater organism samples such as periphyton and invertebrates are often too small in mass to be able to accurately measure their ^210^Po activity concentration. For example, sample size requirements and detection limits for other methods in the literature are summarized in Table 4. The sample quantity requirements and/or detection limits for these methods are not applicable to many environmental sample types. However, the developed method was successful in reducing sample quantities while maintaining sufficiently sensitive detection limits (Fig. 8). Furthermore, applying the CuS microprecipitation method to indirectly measure ^210^Pb not only provided a more sensitive technique, it also allowed the same sample to be used for both the ^210^Po and ^210^Pb analyses, which reduced field sampling efforts and laboratory processing times.

**Table 4:** Overview of analytical methods for determining ^210^Po in environmental samples.

Sample type	Quantity	Analytical protocol	Minium detection limit	References
Water	0.5–1 L	Po plating on silver disk α spectrometry	2 mBq L^−1^	IAEA^32^
Seawater	20 L	Fe(OH)_3_ precipitation Po plating on copper foil α spectrometry	0.004 mBq·L^−1^	Biggin et el.^33^
Fish	20 g (wet weight)	Po plating on silver disk α spectrometry	0.1 mBq·g^−1^	Sadi et al.^34^
Mussels	0.5–3.5 g (dry weight)	Po plating on silver disk α spectrometry	–	Khan et al.^35^
Water (seawater and freshwater)	1 L	Ti(OH)_3_ co-precipitation CuS micro-precipitation α spectrometry	0.02 mBq·L^−1^ (48 h count)	Current method
Biota samples	1–2 g (wet weight)	CuS micro-precipitation α spectrometry	0.04–0.19 mBq·g^−1^ (48 h count)	Current method

## Conclusion

Large data gaps exist in understanding the behavior of the ^210^Po and ^210^Pb in the environment, and this is largely due to challenges associated with measuring these radionuclides at the low-level activity concentrations typically encountered in the environment^14,18^. The developed method is simple and highly sensitive (MDA ~ 0.01 mBq L^−1^). Consistent and high chemical recoveries (> 80%) for abiotic and biotic environmental samples were obtained, thereby making it possible to adequately study the behavior of ^210^Po and ^210^Pb in the environment.

### Supplementary Information

Supplementary Information.

## Data Availability

The ^210^Po and ^210^Pb dataset generated during this research is being prepared for a separate manuscript as part of a doctoral thesis. The datasets generated during the current study are available from the corresponding author on reasonable request.
