# Supplementary material for: A sensitive method to determine 210Po and 210Pb in environmental samples by alpha spectrometry using CuS micro-precipitation
Source: Sci Rep. 2023 Nov 13;13:19754. doi: 10.1038/s41598-023-46230-9 (PMC10643654; doi:10.1038/s41598-023-46230-9)
Supplement: Supplementary file 1 — Supplementary Information. [file 41598_2023_46230_MOESM1_ESM.docx]

**Supplemental Materials**

A sensitive method to determine ^210^Po and ^210^Pb in environmental samples by alpha spectrometry using CuS micro-precipitation

Stephanie Walsh^1^*, Matthew J. Bond^1^, Nicolas Guérin^1^, Jules M. Blais^2^, David J. Rowan^1^

^1^ Canadian Nuclear Laboratories, Chalk River Laboratories, Chalk River, ON, Canada, K0J 1J0

^2^ Department of Biology, University of Ottawa, Ottawa, ON, Canada, K1N 6N5

**Corresponding author:** Stephanie Walsh, Stephanie.Walsh@cnl.ca


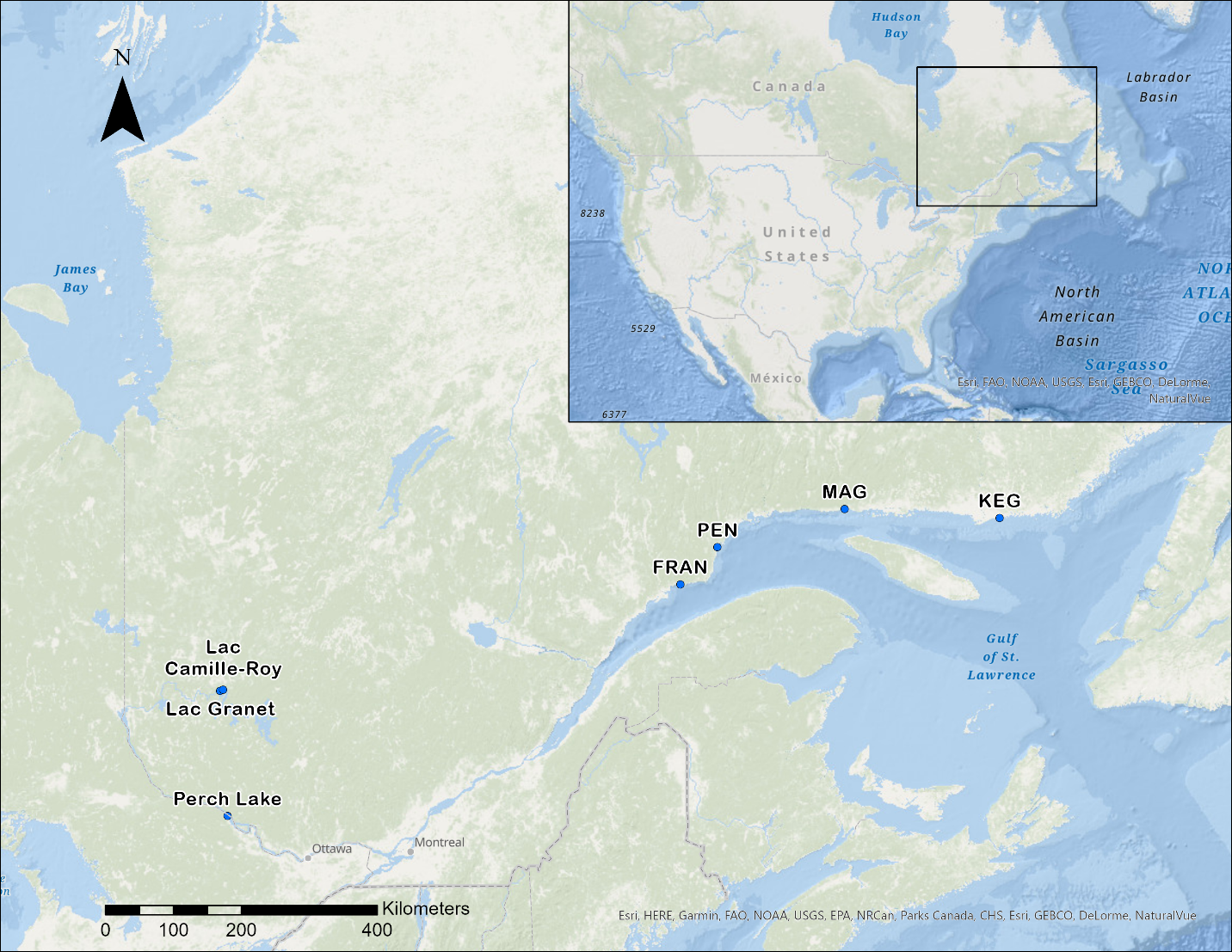


**Supplementary figure S1. Locations of sampling locations for method application and optimization**


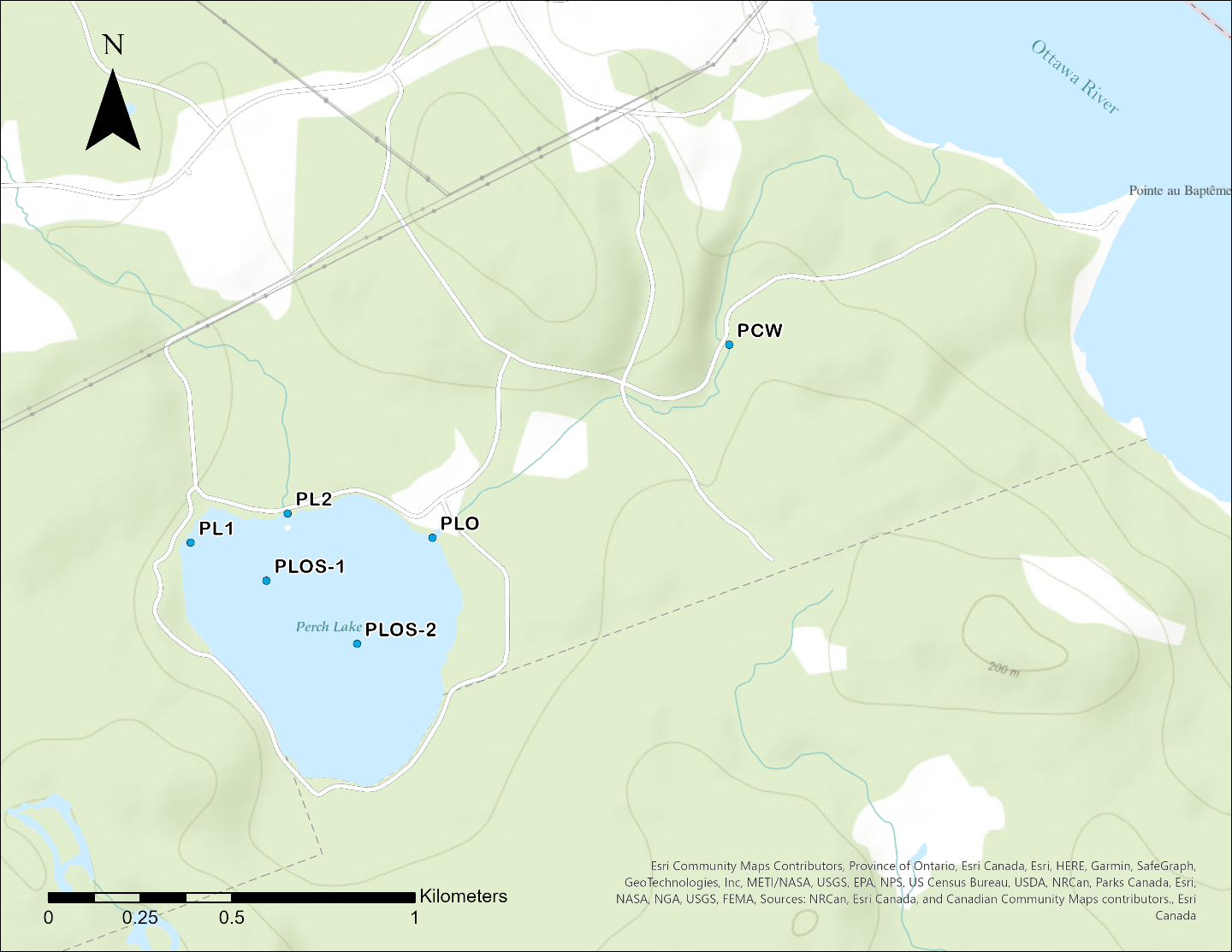


**Supplementary figure S2. Map of surface fresh water sampling locations on Perch Lake on the Chalk River Laboratories site**

**
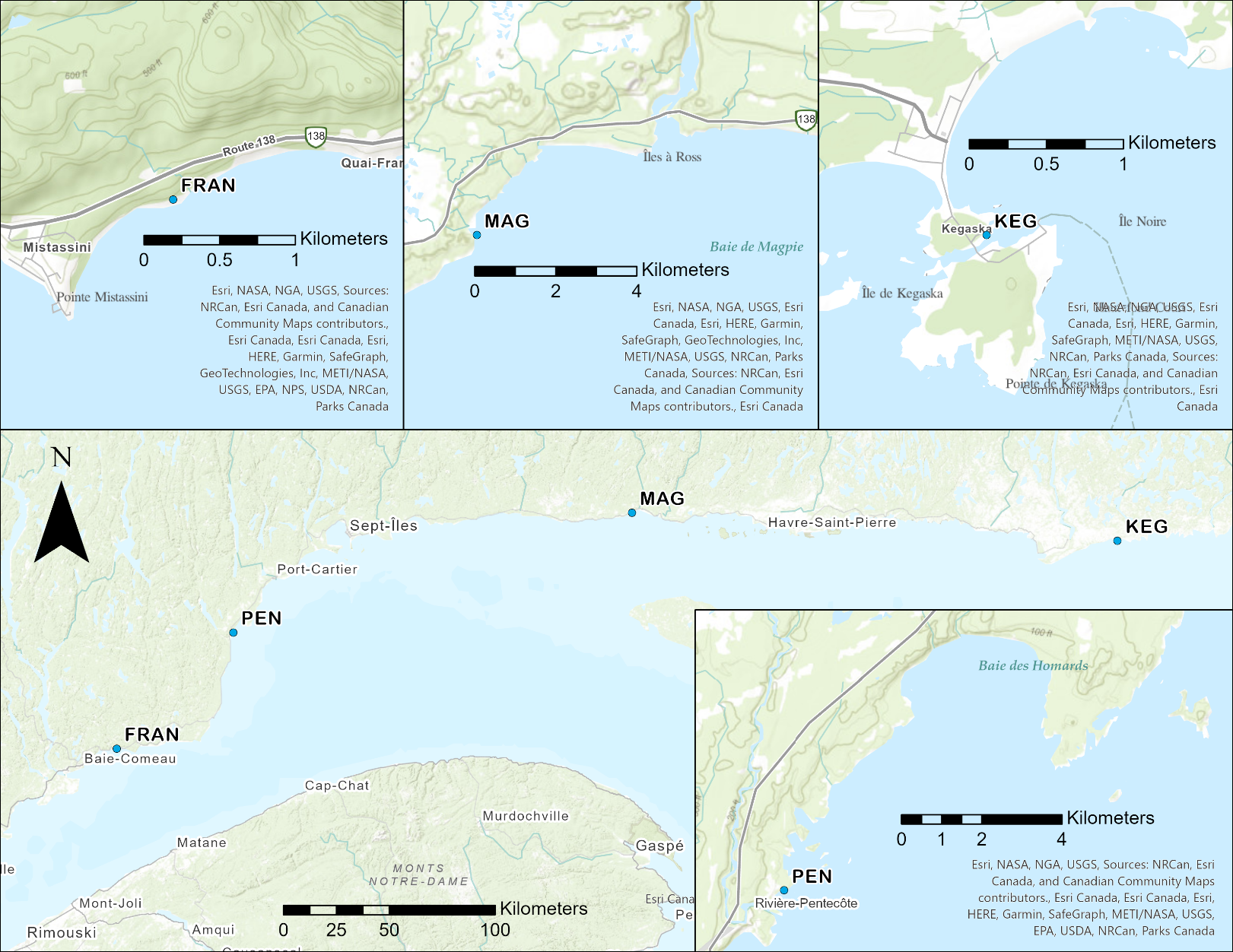
**

**Supplementary figure S3. Map of surface marine water sampling locations along the North Shore of the St. Lawrence**

**
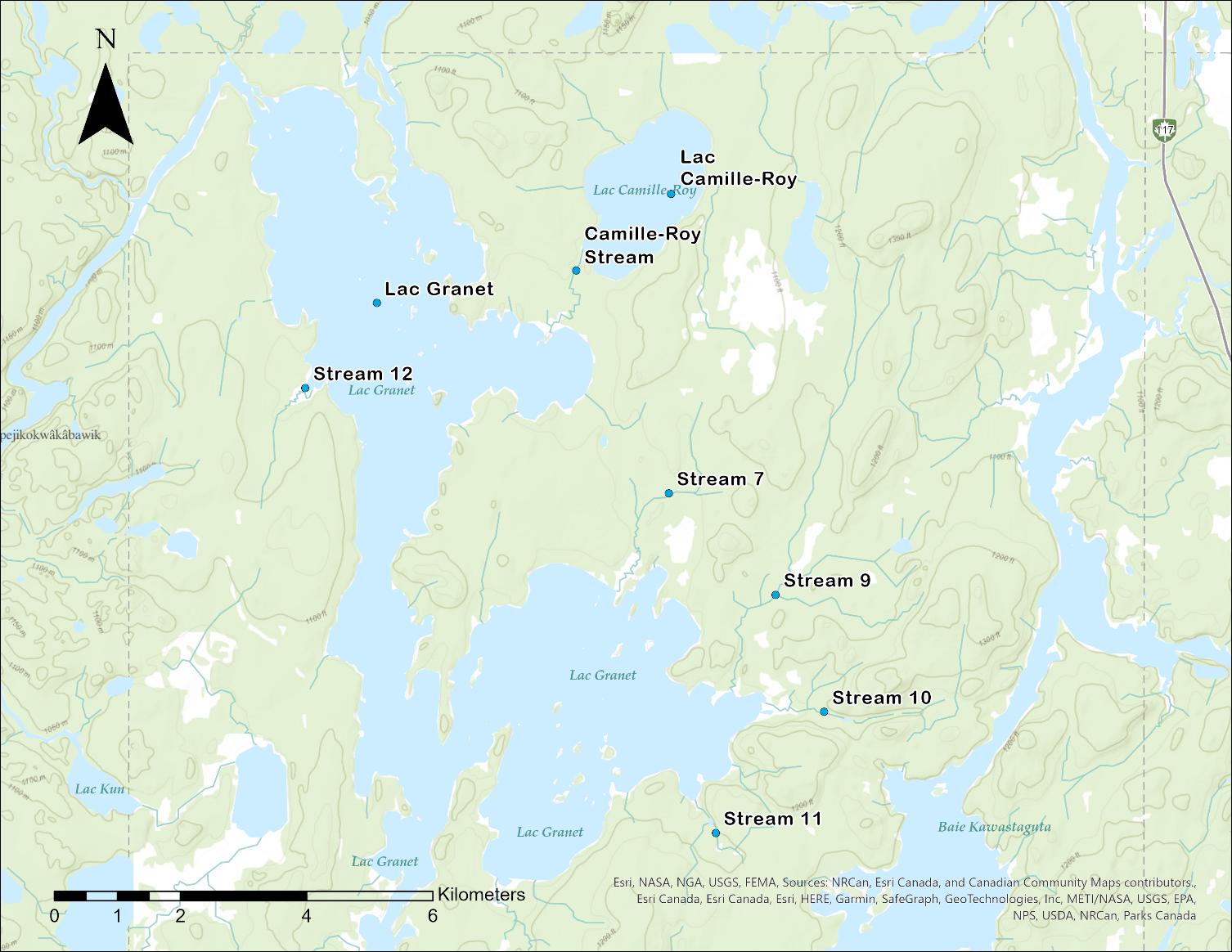
**

**Supplementary figure S4. Map of sampling locations on the Lac Granet and Lac Camille-Roy systems for sediment, particle and biota samples**

**Supplementary table S1. Summary of surface water sampling locations**

| **Site I.D** | **Coordinates** | **Main waterbody** | ***n*** | **Description** |
| --- | --- | --- | --- | --- |
| PLO | 46.035543, -77.371866 | Perch Lake | 4 | Freshwater, lake outflow, nearshore |
| PL1 | 46.035614, -77.379020 | Perch Lake | 5 | Freshwater, lake inlet, nearshore |
| PL2 | 46.036332, -77.375601 | Perch Lake | 1 | Freshwater, lake inlet 2, nearshore |
| PCW | 46.042502, -77.354342 | Perch Lake | 1 | Freshwater, lake outflow creek, nearshore |
| PLOS-1 | 46.034685, -77.376351 | Perch Lake | 1 | Freshwater, offshore |
| PLOS-2 | 46.033447, -77.376330 | Perch Lake | 1 | Freshwater, offshore |
| FRAN | 49.288516, -67.928211 | St. Lawrence River | 1 | Saltwater, nearshore |
| PEN | 49.790779, -67.152387 | St. Lawrence River | 1 | Saltwater, nearshore |
| MAG | 50.303171, -64.499944 | St. Lawrence River/Gulf of St. Lawrence | 1 | Saltwater, nearshore |
| KEG | 50.183595, -61.26831 | Gulf of the St. Lawrence | 1 | Saltwater, nearshore |

**Supplementary table S2. Summary of physiochemical properties measured in surface water samples. Error bars associated with samples PLO (*n* = 4) and PL1 (*n*=5) are standard deviations from the calculated mean, error bars associated with all other samples (*n*=1) are associated with analytical uncertainty**

| **Analyte** | | **Site I.D** | | | | | | | | | |
| --- | --- | --- | --- | --- | --- | --- | --- | --- | --- | --- | --- |
|  |  | **PLO*** | **PL1*** | **PL2** | **PCW** | **PLOS-1** | **PLOS-2** | **MAG** | **FRAN** | **KEG** | **PEN** |
| **DOC** | mg⸱L^-1^ C | 10.88 ± 0 | 12.00 ± 1.51 | 16.02 | 10.26 | N/A | N/A | 0.32 | 0.21 | 0.16 | 0.41 |
| **DIC** | mg⸱L^-1^ C | 3.27 ± 0.44 | 4.05 ± 0.98 | 2.37 | 4.062 | N/A | N/A | 13.09 | 22.03 | 23.51 | 19.42 |
| **pH** |  | 6.49 ± 0.33 | 6.50 ± 0 | 6.00 | 6.5 | 6.25 | 6.50 | N/A | N/A | N/A | N/A |
| **Cond** | µS⸱cm^-1^ | 133 ± 18.24 | 115.5 ± 0 | 115.50 | 115.5 | 115.50 | 115.50 | 29200 | 47100 | N/A | 42200 |
| **Na^+^** | µg⸱mL^-1^ | 14.65 ± 0.35 | 18.55 ± 2.05 | 17.7 ± 0.9 | 13.6 ± 0.7 | N/A | N/A | 4800 ± 200 | 8200 ± 400 | 8900 ± 400 | 7100 ± 400 |
| **K^+^** | µg⸱mL^-1^ | 1.05 ± 0.10 | 1.40 ± 0.02 | 0.86 ± 0.04 | 1.01 ± 0.05 | N/A | N/A | 181 ± 9 | 320 ± 20 | 450 ± 20 | 280 ± 10 |
| **Mg^2+^** | µg⸱mL^-1^ | 2.10 ± 0.40 | 2.20 ± 0.28 | 2.1 ± 0.1 | 1.57 ± 0.08 | N/A | N/A | 600 ± 30 | 1030 ± 50 | 1110 ± 60 | 900 ± 40 |
| **Ca^2+^** | µg⸱mL^-1^ | 4.95 ± 0.50 | 5.10 ± 0.99 | 4.7 ± 0.2 | 5.1 ± 0.3 | N/A | N/A | 210 ± 10 | 320 ± 20 | 330 ± 20 | 280 ± 10 |
| **Cl^-^** | mg⸱L^-1^ | 35.75 ± 0.07 | 43.00 ± 3.96 | 40.75 | 32.9 | N/A | N/A | 14775.79 | 28945.83 | 26938.42 | 23215.25 |
| **NO_3_^-^** | mg⸱L^-1^ | 0.06 | <0.04 | 0.05 | 0.053 | N/A | N/A | N/A | N/A | N/A | N/A |
| **N** | mg⸱L^-1^ | 0.01 | <0.01 | 0.01 | 0.01 | N/A | N/A | N/A | N/A | N/A | N/A |
| **PO_4_^-^** | mg⸱L^-1^ | <0.07 | <0.07 | <0.07 | <0.07 | N/A | N/A | N/A | N/A | N/A | N/A |
| **SO_4_^-^** | mg⸱L^-1^ | 2.91 ± 0,05 | 1.77 ± 0.37 | 1.05 | 3.37 | N/A | N/A | 1477.36 | 2845.61 | 2685.36 | 2270.73 |
| **Pb** | ng⸱mL^-1^ | 0.31 ± 0.02 | 1.25 ± 1.48 | 0.61 | 0.32 | N/A | N/A | 1.2 ± 0.1 | 0.35 ± 0.08 | 0.47 ± 0.08 | 0.48 ± 0.08 |
| **U** | ng⸱mL^-1^ | <0.02 | 0.029 ± 0.008 | 0.021 ± 0.007 | <0.02 | N/A | N/A | 1.60 ± 0.09 | 2.7 ± 0.1 | 2.9 ± 0.1 | 2.3 ± 0.1 |
| **Ba** | ng⸱mL^-1^ | 13.45 ± 0.07 | 15.95 ± 4.03 | 11.9 ± 0.6 | 12.6 ± 0.6 | N/A | N/A | 6.1 ± 0.3 | 7.6 ± 0.4 | 6.7 ± 0.3 | 7.6 ± 0.4 |
| **Ce** | ng⸱mL^-1^ | 1.12 ± 0 | 1.39 ± 0.42 | 1.63 ± 0.08 | 1.17 ± 0.06 | N/A | N/A | 0.094 ± 0.008 | 0.052 ± 0.007 | <0.02 | 0.113 ± 0.009 |
| **Cs** | ng⸱mL^-1^ | 0.007 ± 0.001 | 0.008 ± 0.003 | 0.008 ± 0.001 | 0.0043 ± 0.001 | N/A | N/A | 0.4 ± 0.1 | 0.7 ± 0.1 | 0.6 ± 0.1 | 0.5 ± 0.1 |
| **Mo** | ng⸱mL^-1^ | 0.12 ± 0.03 | 0.07 ± 0.01 | 0.10 ± 0.01 | 0.15 ± 0.03 | N/A | N/A | 5.4 ± 0.3 | 8.9 ± 0.4 | 9.2 ± 0.5 | 7.3 ± 0.4 |
| **Rb** | ng⸱mL^-1^ | 1.65 ± 0.08 | 2.55 ± 0.21 | 1.27 ± 0.06 | 1.51 ± 0.08 | N/A | N/A | 53 ± 3 | 86 ± 4 | 90 ± 5 | 74 ± 4 |
| **Sr** | ng⸱mL^-1^ | 39.0 ± 1.4 | 47.0 ± 8.5 | 35 ± 2 | 41 ± 2 | N/A | N/A | 3700 ± 200 | 6200 ± 300 | 6400 ± 300 | 5300 ± 300 |

**Supplementary table S3. Inventory of abiotic and biotic samples collected in Lac Granet and Lac Camille-Roy**

| **Sample ID** | **Water Body Name** | **Sample Type** | **Sample Taxa / Note** | **Collection Date** | **GPS Coordinates** | |
| --- | --- | --- | --- | --- | --- | --- |
|  |  |  |  |  | **Northing** | **Easting** |
| CR-S-Sed | Lac Camille-Roy (stream) | Sediment | - | 10/08/2021 | 47.819806 | -77.481514 |
| LG-S7-Sed | Lac Granet, Stream 7 | Sediment | - | 10/08/2021 | 47.823064 | -77.494317 |
| LG-S9-Sed | Lac Granet, Stream 9 | Sediment | - | 10/08/2021 | 47.819223 | -77.482163 |
| LG-S11-Sed | Lac Granet, Stream 11 | Sediment | - | 10/08/2021 | 47.739860 | -77.452011 |
| LG-S7-P | Lac Granet, Stream 7 | Particles | - | 10/08/2021 | 47.823064 | -77.494317 |
| LG-S9-P | Lac Granet, Stream 9 | Particles | - | 10/08/2021 | 47.819223 | -77.482163 |
| LG-S7-Algae | Lac Granet, Stream 7 | Algae | - | 10/08/2021 | 47.823064 | -77.494317 |
| LG-S10-Algae | Lac Granet, Stream 10 | Algae | - | 10/08/2021 | 47.757076 | -77.429069 |
| LG-S11-Algae | Lac Granet, Stream 11 | Algae | - | 10/08/2021 | 47.739860 | -77.452011 |
| LG-S7-Mac | Lac Granet, Stream 7 | Macrophyte | Sedge (Carex sp.) | 10/08/2021 | 47.823064 | -77.494317 |
| LG-S7-Bay-Mac | Lac Granet, Stream 7 bay | Macrophyte | Sedge (Carex sp.) | 11/08/2021 | 47.812725 | -77.495661 |
| LG-S9-Mac | Lac Granet, Stream 9 | Macrophyte | Sedge (Carex sp.) | 10/08/2021 | 47.819223 | -77.482163 |
| LG-S10-Mac | Lac Granet, Stream 10 | Macrophyte | Sedge (Carex sp.) | 10/08/2021 | 47.757076 | -77.429069 |
| LG-Mac-21-1 | Lac Granet, main lake | Macrophyte | Potamogeton (Potamogetonaceae) | 12/08/2021 | 47.815187 | -77.523737 |
| LG-Mac-21-2 | Lac Granet, main lake | Macrophyte | Sedge (Carex sp.) | 12/08/2021 | 47.815187 | -77.523737 |
| LG-Plank-21-1 | Lac Granet, main lake | Plankton | Phytoplankton & Zooplankton mix | 11/08/2021 | 47.815187 | -77.523737 |
| LG-Plank-21-2 | Lac Granet, main lake | Plankton | Phytoplankton & Zooplankton mix | 11/08/2021 | 47.815187 | -77.523737 |
| CR-S-Od | Camille-Roy, Stream | Invertebrate | Dragonfly nymph Odonata (Gomphidae) | 10/08/2021 | 47.819806 | -77.481514 |
| CR-S-Beetle | Camille-Roy, Stream | Invertebrate | Water scavenger beetle (Hydrophilidae) | 10/08/2021 | 47.819806 | -77.481514 |
| CR-S-Cad | Camille-Roy, Stream | Invertebrate | Caddisfly (Trichoptera) | 10/08/2021 | 47.819806 | -77.481514 |
| LG-S7-Cad | Lac Granet, Stream 7 | Invertebrate | Caddisfly (Trichoptera) | 10/08/2021 | 47.823064 | -77.494317 |
| LG-S7-Beetle (mix) | Lac Granet, Stream 7 | Invertebrate | Water scavenger beetle (Hydrophilidae) | 10/08/2021 | 47.823064 | -77.494317 |
| LG-S7-Mouth-Od | Lac Granet, Stream 7 mouth | Invertebrate | Dragonfly nymph Odonata (Gomphidae) | 11/08/2021 | 47.813808 | -77.495135 |
| LG-S7-Mouth-Dam | Lac Granet, Stream 7 mouth | Invertebrate | Damselfly (Zygoptera) | 11/08/2021 | 47.813808 | -77.495135 |
| LG-S7-Mouth-Beetle | Lac Granet, Stream 7 mouth | Invertebrate | Water scavenger beetle (Hydrophilidae) | 11/08/2021 | 47.813808 | -77.495135 |
| LG-S9-Cad | Lac Granet, Stream 9 | Invertebrate | Caddisfly (Trichoptera) | 10/08/2021 | 47.819223 | -77.482163 |
| LG-S9-Beetle | Lac Granet, Stream 9 | Invertebrate | Water scavenger beetle (Hydrophilidae) | 10/08/2021 | 47.819223 | -77.482163 |
| LG-S9-Od | Lac Granet, Stream 9 | Invertebrate | Dragonfly nymph Odonata (Gomphidae) | 10/08/2021 | 47.819223 | -77.482163 |
| LG-S10-Od | Lac Granet, Stream 10 | Invertebrate | Dragonfly nymph Odonata (Gomphidae) | 10/08/2021 | 47.757076 | -77.429069 |
| LG-S10-Dam | Lac Granet, Stream 10 | Invertebrate | Damselfly (Zygoptera) | 10/08/2021 | 47.757076 | -77.429069 |
| LG-S10-Cad | Lac Granet, Stream 10 | Invertebrate | Caddisfly (Trichoptera) | 10/08/2021 | 47.757076 | -77.429069 |
| LG-Bay-Od-21 | Lac Granet, main lake bay | Invertebrate | Dragonfly nymph Odonata (Gomphidae) | 10/08/2021 | 47.822942 | -77.513091 |
| LG-DH-Oligo-21-1 | Lac Granet, main lake deep hole | Invertebrate | Tubifex (Oligochaeta) | 11/08/2021 | 47.814410 | -77.528118 |
| LG-DH-Hex-21-1 | Lac Granet, main lake deep hole | Invertebrate | Hexagenia (Ephemeridae) | 11/08/2021 | 47.814410 | -77.528118 |
| LG-S12-Bay-clam flesh | Lac Granet, stream 12 bay | Invertebrate (mollusc) | Eastern elliptio (Unionidae) | 10/08/2021 | 47.806883 | -77.534775 |
| LG-S12-Bay-clam shell | Lac Granet, stream 12 bay | Invertebrate (mollusc) | Eastern elliptio (Unionidae) | 10/08/2021 | 47.806883 | -77.534775 |
| LG-DH-21-1-clam flesh | Lac Granet, main lake deep hole | Invertebrate (mollusc) | Eastern elliptio (Unionidae) | 11/08/2021 | 47.814410 | -77.528118 |
| LG-DH-21-1-clam shell | Lac Granet, main lake deep hole | Invertebrate (mollusc) | Eastern elliptio (Unionidae) | 11/08/2021 | 47.814410 | -77.528118 |
| LG-DH-21-1-snail flesh | Lac Granet, main lake deep hole | Invertebrate (mollusc) | Pond snail (Lymnaeidae) | 11/08/2021 | 47.814410 | -77.528118 |
| LG-DH-21-1-snail shell | Lac Granet, main lake deep hole | Invertebrate (mollusc) | Pond snail (Lymnaeidae) | 11/08/2021 | 47.814410 | -77.528118 |
| CR-S-BF | Camille-Roy, stream | Prey fish | Fine scale dace (Chrosomus neogaeus) | 10/08/2021 | 47.819806 | -77.481514 |
| LG-BF-21-1 | Lac Granet, main lake | Prey fish | Yellow perch (Percidae) | 12/08/2021 | 47.823746 | -77.512583 |
| LG-BF-21-2 | Lac Granet, main lake | Prey fish | Finescale dace (Chrosomus neogaeus) | 12/08/2021 | 47.823746 | -77.512583 |
| LG-BF-21-3 | Lac Granet, main lake | Prey fish | Emerald shiner (Notropis atherinoides) | 12/08/2021 | 47.823746 | -77.512583 |
| LG-Wall-21-1-tissue | Lac Granet, main lake | Predator fish | Walleye (Sander vitreus) | 11/08/2021 | 47.815187 | -77.523737 |
| LG-Wall-21-1-muscle | Lac Granet, main lake | Predator fish | Walleye (Sander vitreus) | 11/08/2021 | 47.815187 | -77.523737 |
| LG-Wall-21-2-tissue | Lac Granet, main lake | Predator fish | Walleye (Sander vitreus) | 11/08/2021 | 47.815187 | -77.523737 |
| LG-Wall-21-2-muscle | Lac Granet, main lake | Predator fish | Walleye (Sander vitreus) | 11/08/2021 | 47.815187 | -77.523737 |
| LG-Wall-21-3-tissue | Lac Granet, main lake | Predator fish | Walleye (Sander vitreus) | 12/08/2021 | 47.815187 | -77.523737 |
| LG-Wall-21-3-muscle | Lac Granet, main lake | Predator fish | Walleye (Sander vitreus) | 12/08/2021 | 47.815187 | -77.523737 |
| LG-Wall-21-4-muscle | Lac Granet, main lake | Predator fish | Walleye (Sander vitreus) | 12/08/2021 | 47.815187 | -77.523737 |
| LG-Wall-21-5-tissues | Lac Granet, main lake | Predator fish | Walleye (Sander vitreus) | 12/08/2021 | 47.815187 | -77.523737 |
| LG-Wall-21-5-muscle | Lac Granet, main lake | Predator fish | Walleye (Sander vitreus) | 12/08/2021 | 47.815187 | -77.523737 |
| LG-Npike-21-1-tissue | Lac Granet, main lake | Predator fish | YOY Northern pike (Esox lucius) | 11/08/2021 | 47.815187 | -77.523737 |
| LG-Npike-21-1-muscle | Lac Granet, main lake | Predator fish | YOY Northern pike (Esox lucius) | 11/08/2021 | 47.815187 | -77.523737 |
| LG-Npike-21-3-tissue | Lac Granet, main lake | Predator fish | YOY Northern pike (Esox lucius) | 12/08/2021 | 47.815187 | -77.523737 |
| LG-Npike-21-3-muscle | Lac Granet, main lake | Predator fish | YOY Northern pike (Esox lucius) | 12/08/2021 | 47.815187 | -77.523737 |
